# Sir A. Cooper on Fungoid Disease of the Testis

**Published:** 1831-01-01

**Authors:** 


### FUNGOID DISEASE OF THE TESTIS.

IN our review of Sir Astley Cooper's work on the Diseases of the Testis, we omitted the details of several cases, with the view of reserving them for this department of the Journal. Three instances of the fungoid disease remain to be noticed here.

*Case* 1. On Dec. 7th, 1807, Sir Astley removed the testicle of Mr. A——, of Worcester, for this disease. For a fortnight he proceeded tolerably well, but then an abscess formed in the course of the cord, and a fungous appearance presented itself at its extremity, when the ligature had been secured. By the 11th of January, 1808, the wound was quite healed, and the patient went into the country. Here he gained 13lbs. in weight, but he returned to town on the 1st of February, and complained of slight pain in his abdomen. On the 10th, there was observed a swelling of the scrotum, and on pressure there issued a brain-like pulpy substance, of brownish red colour; the swelling subsided, but the pain in the abdomen remained. By the 21st, a fungous tumour had agained formed in the scrotum, and between the edges of the wound. It grew rapidly, the ulcer had everted edges and a fungous surface, and a hard swelling surrounded it. He had pain in his stomach, and was as sallow as before the operation. On the 1st of March, a fungus had arisen with rapidity opposite the end of the cord. Sir Astley removed it, but with difficulty, as it adhered to the tunica vaginalis of the other testis, and on that side he had a hernia. Pain in the stomach now became severe, and a tumour could soon be perceived on the right side of the abdomen, above the level of the navel. The pain was only relieved by opium. On the 15th, he was attacked with violent pain of the abdomen and vomiting, and after this he was often sick, had cardialgia, and indigestion; the pulse was 130 in the minute. He was removed to Islington, but the symptoms continued to make progress. A swelling of the leg and thigh of the affected side now took place; he vomited frequently, had hiccough, and was much reduced. Diarrhœa set in, the symptoms of sinking made progress, and on the 20th of May he died. Towards the latter part of his existence, this unfortunate patient had frequently rigors succeeded by heat, and the left leg began to swell a short time before his death. In the morning of the day he died he became quite easy, and even when his extremities were cold, he said that he felt as if he could walk down stairs. The mind was energetic to the last.

*Dissection.* The spermatic cord did not seem to be diseased. Behind the duodenum was a tumour, to which the intestine adhered; to the posterior part of the tumour the aorta and vena cava were attached. The tumour was as large as the head of a child. In parts it was white and fibrous, in others it resembled brain in different degrees of putrefaction: when the more solid portion was compressed, there issued a fluid like cream tinged with blood. Many of the mesenteric glands were enlarged. The aorta and vena cava were greatly diseased, each of them being tuberculated, and the aorta nearly closed by a tuberculous secretion. The thoracic duct was unaffected.

*Case* 2. Mr. ——, aged 46, of Ramsbury, Wilts, presented himself to Sir Astley in December, 1807, with the right testis treble its natural size, and as hard as marble. It was only painful when pressed, but felt heavy, and the swelling was increased by exercise. He had some pain in the groin, looked sallow, and had a fixed blush in the centre of the cheek. The disease had commenced three or four months previously, with an uneasy sensation in the testicle on touching its upper part. It then began to grow large and hard, and feel heavy. The pain in the groin had only existed a few days.

On the following day Sir Astley amputated the testis, the wound healed quickly, and in January he returned to the country, apparently in good health. On examination of the testicle, the body and epididymis were found in a pulpy state, and filled with soft fibrine, mostly of yellowish white colour, but coloured in parts by blood. The hardness of the testis had arisen from the excessive distention of the tunica albuginea. The tumour was very unequally organized. Quicksilver thrown in by the vas deferens proceeded as far as the beginning of the epididymis, but would pass no farther. In a few months after this person's return into the country he died of vomiting, swelling of the legs and thighs, violent pain in the abdomen, hiccup, sallow countenance, abdominal tumour, and great tenderness on pressure of the abdomen.

*Case* 3. In March 1823, a fungous testis was removed from Mr. E. a baker of Cambridge, 25 years of age. He had tried various remedies, and amongst them mercury, without benefit. In about three weeks after the operation he was able to resume his business. In the following November he began to complain of pain in his back, extending forwards to the abdomen, on the side from which the testicle had been removed. In a few days it had increased greatly, and was attended with vomiting and fever, which were subdued by two copious bleedings from the arm and aperient medicine. In about a fortnight the symptoms re-appeared, and some fulness in the affected side of the abdomen was discovered. The tumour continued to increase until May, 1824, when the tincture of iodine, in large doses, was tried for some weeks. He derived no benefit from this remedy, was obliged to have recourse to opium, and died in the following July.

On opening the abdomen, large irregular masses of a soft pulpy structure presented themselves in every direction, though evidently springing from that side from which the testicle had been removed, and having attachments posterior to the peritoneum. The texture of the morbid growth was so loose that a handful of it might be scooped up without using any force.

